# Physiological and Molecular Characterization of Hydroxyphenylpyruvate Dioxygenase (HPPD)-inhibitor Resistance in Palmer Amaranth (*Amaranthus palmeri* S.Wats.)

**DOI:** 10.3389/fpls.2017.00555

**Published:** 2017-04-11

**Authors:** Sridevi Nakka, Amar S. Godar, Prashant S. Wani, Curtis R. Thompson, Dallas E. Peterson, Jeroen Roelofs, Mithila Jugulam

**Affiliations:** ^1^Department of Agronomy, Kansas State University, ManhattanKS, USA; ^2^Department of Plant Sciences, University of California, DavisCA, USA; ^3^Division of Biology, Kansas State University, ManhattanKS, USA

**Keywords:** mesotrione, resistant mechanism, target-site, non-target-site, metabolism, absorption and translocation, HPPD expression

## Abstract

Herbicides that inhibit hydroxyphenylpyruvate dioxygenase (HPPD) such as mesotrione are widely used to control a broad spectrum of weeds in agriculture. *Amaranthus palmeri* is an economically troublesome weed throughout the United States. The first case of evolution of resistance to HPPD-inhibiting herbicides in *A. palmeri* was documented in Kansas (KS) and later in Nebraska (NE). The objective of this study was to investigate the mechansim of HPPD-inhibitor (mesotrione) resistance in *A. palmeri.* Dose response analysis revealed that this population (KSR) was 10–18 times more resistant than their sensitive counterparts (MSS or KSS). Absorbtion and translocation analysis of [^14^C] mesotrione suggested that these mechanisms were not involved in the resistance in *A. palmeri*. Importantly, mesotrione (>90%) was detoxified markedly faster in the resistant populations (KSR and NER), within 24 hours after treatment (HAT) compared to sensitive plants (MSS, KSS, or NER). However, at 48 HAT all populations metabolized the mesotrione, suggesting additional factors may contribute to this resistance. Further evaluation of mesotrione-resistant *A. palmeri* did not reveal any specific resistance-conferring mutations nor amplification of *HPPD* gene, the molecular target of mesotrione. However, the resistant populations showed 4- to 12-fold increase in *HPPD* gene expression. This increase in *HPPD* transcript levels was accompanied by increased HPPD protein expression. The significant aspects of this research include: the mesotrione resistance in *A. palmeri* is conferred primarily by rapid detoxification (non-target-site based) of mesotrione; additionally, increased *HPPD* gene expression (target-site based) also contributes to the resistance mechanism in the evolution of herbicide resistance in this naturally occurring weed species.

## Introduction

Mesotrione is a synthetic triketone herbicide chemically known as 2-[4-(methylsulfonyl)-2-nitrobenzoyl]-1,3-cyclohexanedione and biochemically inhibits 4-hydroxyphenylpyruvate dioxygenase (HPPD). This enzyme is important in the catabolism of tyrosine and anabolism of plastoquinones, tocopherols, and subsequently carotenoid biosynthesis (Beaudegnies et al., 2009). Plastoquinone plays a vital role in two significant pathways: (a) as an essential component of photosynthetic electron transfer from photosystem II (PS II) to photosystem I in the process of generating ATP, and (b) acts as an important cofactor for phytoene desaturase, a key enzyme in the carotenoid biosynthesis pathway. Carotenoids are light harvesting molecules, and protect plants from photo oxidation by quenching the triplet chlorophyll and prevent the formation of destructive singlet oxygen (Siefermann, 1987).

4-hydroxyphenylpyruvate dioxygenase-inhibitors are a relatively new class of chemistry discovered about three decades ago and are widely used in agriculture for weed management. HPPD-inhibitors are broadly classified into three chemical families: isoxazoles (e.g., isoxaflutole and pyrasulfotole), pyrazolones (e.g., topramezone), and triketones (e.g., mesotrione and tembotrione) depending on the chemical structure and properties (Lee et al., 1998). Upon treatment with these herbicides, susceptible plants exhibit characteristic bleaching symptoms as a result of loss of carotenoid synthesis and eventually leading to lipid peroxidation of cell membranes. Mesotrione is one of the most widely used HPPD-inhibiting herbicides that selectively control many broad-leaved weeds, including *Amaranthus palmeri*, and some grasses in corn (*Zea mays* L.) when applied post as well as pre-emergence herbicide (Mitchell et al., 2001). Rapid metabolism, via ring hydroxylation mediated by cytochrome P450 monooxygenase(s) combined with reduced absorption of mesotrione has been attributed to selectivity of this herbicide in corn (Mitchell et al., 2001). The differential selectivity of mesotrione and many herbicides such as sulfonylureas (ALS-inhibitors) and triazines (PS II-inhibitors) between crops and weeds is attributed to the ability of the crops to rapidly detoxify these compounds by cytochrome P450 monooxygenases or glutathione *S*-transferases (GSTs) (Hawkes et al., 2001). On the other hand, the differential selectivity of mesotrione between monocot and dicot species is attributed to HPPD enzyme in monocots being less sensitive to the inhibitors. Tobacco, a dicot species, is highly sensitive to mesotrione, however, when transformed with a *HPPD* gene from wheat, showed tolerance to this herbicide (Hawkes et al., 2001). Transgenic soybeans tolerant to mesotrione, tembotrione, and isoxaflutole have been developed with an herbicide-insensitive maize HPPD to increase the selectivity and spectrum of weed control (Siehl et al., 2014). Mesotrione and other HPPD-inhibitors are important in controlling several ALS- and PS II-inhibitor resistant weed biotypes (Sutton et al., 2002). It is also important to preserve the effectiveness and extend the use of these herbicides as no herbicides with new modes of action have been introduced in the last 20 years (Duke, 2012), and new herbicide-resistant traits are being stacked in crops to control weeds.

Palmer amaranth (*A. palmeri* S. Wats.) is one of the most economically important weeds in corn, soybean (*Glycine max* L.), cotton (*Gossypium* spp.), sorghum (*Sorghum bicolor* L.), and many other cropping systems throughout the United States (Ward et al., 2013; Chahal et al., 2015). Infestation of Palmer amaranth can significantly decrease the quality, and cause huge yield losses ranging from 63 to 91% depending on the density and duration of interference in different crops (Ward et al., 2013). Management of Palmer amaranth is possible using several herbicide chemistries, however, repeated and extensive use of herbicides resulted in the evolution of resistance to multiple herbicides with various modes of action such as 5-enolpyruvylshikimate-3-phosphate synthase (EPSPS)-, acetolactate synthase (ALS)-, PS II-, microtubule-, more recently to protoporphyrinogen oxidase (PPO)- and HPPD-inhibitor herbicides (Heap, 2017). Currently, two weed species in the Amaranthaceae family, common waterhemp (*A. tuberculatus*) and Palmer amaranth, have evolved resistance to several HPPD-inhibiting herbicides which offer a feasible option to manage other herbicide-resistant weeds including glyphosate-resistant Palmer amaranth (Norsworthy et al., 2008). HPPD-inhibitor resistant waterhemp was first reported in Illinois (IL) in 2009 (Hausman et al., 2011). Detoxification mediated by cytochrome P450 monooxygenases has been reported to confer mesotrione resistance in this waterhemp population (Ma et al., 2013).

In central Kansas (KS), a Palmer amaranth population with resistance to HPPD-inhibitors was first documented in Stafford County and subsequently confirmed in 2012 (Thompson et al., 2012). Later, HPPD-inhibitor resistant Palmer amaranth populations were also found in the nearby state of Nebraska (NE) in a corn field, which had a history of continuous use of HPPD-inhibitors (Sandell et al., 2012). Interestingly, the field in KS where HPPD-inhibitor-resistant Palmer amaranth was found, had no previous history of applications of HPPD-inhibitors, but did have a long history of PS II- and ALS-inhibiting herbicides. This population was initially found resistant to Huskie^®^ (Bayer Crop Science), a mixture of pyrasulfotole (HPPD-inhibitor) and bromoxynil (PS II-inhibitor) and is also resistant to several other HPPD-inhibitors such as mesotrione, tembotrione, and topramezone and was also found to be resistant to atrazine, a widely used PS-II inhibitor (Lally et al., 2010; Thompson et al., 2012). The mechanism of HPPD-inhibitor resistance in the Palmer amaranth populations from KS or NE is unknown. The objectives of this research were to determine the mechanism(s) of resistance to mesotrione in the HPPD-inhibitor resistant Palmer amaranth populations from KS and NE.

## Materials and Methods

### Plant Material and Growth Conditions

Three mesotrione ‘resistant’ Palmer amaranth populations from Kansas (KS) and Nebraska (NE), designated as KSR, KSR2, NER and five mesotrione ‘susceptible’ populations from Mississippi (MS), KS, and NE, designated as MSS, KSS, KSS II, KSS III, and NES, respectively, were used in this study. KSR seed was derived by crossing male and female plants of Palmer amaranth from KSR2 that survived 105 g ai ha^-1^, field use rate of mesotrione (Callisto^TM^, Syngenta Crop Protection) under greenhouse conditions to generate a more homogeneous resistant population. However, KSR2 seed was collected from Palmer amaranth plants which survived a HPPD-inhibitor application in a field in Stafford County, KS (Thompson et al., 2012) that had wheat-sorghum crop rotation. Seed of NER was collected from Palmer amaranth that survived mesotrione application in a corn field in NE (Sandell et al., 2012). NES population is also provided by Sandell et al. (2012) and MSS by Syngenta. The mesotrione-susceptible populations were selected based on their sensitivity to mesotrione at field recommended rate (i.e., completely killed at field rate) relative to resistant populations. The three susceptibles from KS comes from three distinctly separated locations. KSS (Thompson et al., 2012), KSS II (37°31′05.74″ N and 097°29′42.43″ W), KSS III (37°59′24.0″ N and 100°49′12.0″ W) are from fields in Riley, Reno, and Finney Counties in KS, respectively. Seeds of mesotrione-susceptible and -resistant Palmer amaranth were germinated in small trays (25 cm × 15 cm × 2.5 cm) with commercial potting mixture (Miracle Gro). Seedlings 2–3 cm tall, were transplanted into small pots (6 cm × 6 cm × 6.5 cm) in the greenhouse, maintained at 25/20°C and 15/9 h photoperiod, supplemented with 250 μmol m^-2^ s^-1^ illumination provided with sodium vapor lamps. When the plants reached 5–6 cm tall, they were transferred to a growth chambers maintained at 32.5/22.5°C, 15/9 h photoperiod, 60–70% relative humidity. Light in the growth chamber was provided by fluorescent bulbs delivering 550 μmol m^-2^ s^-1^ photon flux at plant canopy level. Plants were watered as needed regularly both under greenhouse as well as growth chamber conditions.

### Mesotrione Dose Response Assay

Mesotrione-resistant (KSR) and -susceptible (MSS and KSS) Palmer amaranth were grown under greenhouse and growth chamber conditions as described above. Initially, the KSR and KSR2 Palmer amaranth populations were screened with the commercial field application rate of 105 g ai ha^-1^ mesotrione to determine the frequency of resistant individuals in the population before determining the level of resistance by dose response assay. The frequency of resistance was 90–95% and 60–70% in KSR and KSR2, respectively (data not shown). For the dose response analysis, when the Palmer amaranth plants (MSS, KSS, and KSR) were 10–12 cm tall with 8–10 leaves, mesotrione was applied at 0, 6.5, 13.125, 26.25, 52.5, 105 (1X), 210, 315, 420, and 840 g ai h^-1^, where 1X represents the field recommended rate of mesotrione. This stage (8–10 leaves) is the phenological stage at which most farmers in KS and NE apply mesotrione to control Palmer amaranth. Required adjuvants, crop oil concentrate (COC, Agridex) and ammonium sulfate (AMS, Liquid N-Pak; Winfield) at 1% v/v and 1% w/v (8.5 lb/100 gal = 1% w/v), respectively, were included, respectively, in all the treatments to enhance droplet-to-leaf surface contact. Treatments were applied with a bench-type track sprayer (Generation III, DeVries Manufacturing, RR 1 Box 184, Hollandale, MN, USA) equipped with a flat-fan nozzle tip (80015LP TeeJet tip, Spraying Systems Co., P.O. Box 7900, Wheaton, IL, USA) delivering 187 L ha^-1^ at 222 kPa in a single pass at 4.8 km h^-1^. Following treatment, plants were returned to the same growth chambers (within 30 min after treatment). Treatments were arranged in a completely randomized design with five replications and the experiment was repeated three times. Treated plants were clipped off at the soil surface and immediately weighed (aboveground fresh biomass) 3 weeks after treatment (WAT). Harvested plants were packed in paper bags and oven (Precision Scientific Thelco Laboratory Oven) dried at 60°C for a week before measuring dry biomass.

### Absorption of [^14^C] Mesotrione and Translocation of [^14^C] Compounds

Greenhouse grown seedlings (as described above) of KSR and MSS and KSS Palmer amaranth were moved to growth chamber 2–3 days before applying [^14^C] mesotrione to allow the plants to acclimate. Ten to twelve centimeters tall (8–10 leaf stage) plants were treated with a total of 3.3 kBq of [phenyl-U-^14^C]-labeled mesotrione with specific activity of 781 M Bq g^-1^. Unlabeled mesotrione was added to the radioactive solution to obtain 105 g ai ha^-1^ mesotrione in a carrier volume of 187 L. Additionally, COC (Agridex) and AMS (Liquid N-Pak; Winfield) were added at 1% v/v and 1% w/v, respectively, to this mixture to enhance droplet-to-leaf surface contact. A total volume of 10 μL was applied as 10 1 μL droplets on the upper surface of the fourth youngest leaf. The treated plants were returned to the same growth chamber. Plants were harvested at 48 and 72 hours after treatment (HAT) and separated into treated leaf (TL), leaves above the treated leaf (ATL), and leaves below the treated leaf (BTL) and wrapped in a single layer of tissue paper. Treated leaves were washed with 5 mL wash solution (10% methanol and 0.05% Tween) for 60 s in a 20 mL scintillation vial to remove any unabsorbed herbicide. Radioactivity in the leaf rinsate was measured using liquid scintillation spectrometry (LSS: Tricarb 2100 TR Liquid Scintillation Analyzer; Packard Instrument Co., Meriden, CT, USA). Plant parts were oven (Precision Scientific Thelco Laboratory Oven) dried at 60°C for 48 h and total radioactivity absorbed was quantified by combusting using a biological oxidizer (OX-501, RJ Harvey Instrument) and LSS. Total [^14^C] mesotrione absorption was determined as; % absorption = (total radioactivity applied – radioactivity recovered in wash solution) × 100/total radioactivity applied. Herbicide translocation was determined as; % translocation = 100 – % radioactivity recovered in treated leaf, where % radioactivity recovered in treated leaf = radioactivity recovered in treated leaf × 100/radioactivity absorbed. Six replications were included in each treatment and the experiment was repeated.

### Metabolism of Mesotrione in Whole Plant and Treated Leaves

KSR, NER and MSS, KSS and NES Palmer amaranth populations were grown as described previously for [^14^C] mesotrione absorption and translocation experiments. Twenty microliter of [^14^C] mesotrione containing 7.2 kBq was applied on 10–12 cm tall (8–10 leaf stage) plants as 10 1μL droplets on the adaxial surface of fully expanded fourth and fifth youngest leaves. [^14^C] mesotrione and its metabolites were extracted as described in Godar et al. (2015). Treated leaves were harvested 4, 8, 16, 24, 48, and 72 HAT and washed with wash solution to remove unabsorbed herbicide. Whole plant tissue including the washed treated leaves or only the treated leaves were then frozen in liquid nitrogen and homogenized using a mortar and pestle. [^14^C] mesotrione and its metabolites were extracted with 15 ml of 90% acetone at 4°C for 16 h. The samples were centrifuged at 5,000 × *g* for 10 min and supernatant from each sample was concentrated at 45°C for 2–3 h with a rotary evaporator (Centrivap, Labconco) until a final volume of 500–1000 μL of extract was reached. The extract was then transferred to a 1.5 mL microcentrifuge tube and centrifuged at high speed (10,000 g) for 10 min at room temperature. The total radioactivity in each sample was measured by LSS and samples were normalized to 0.05 KBq/50 μL (3000 dpm/50 μL) amount of [^14^C]-labeled compounds by diluting the samples with acetonitrile:water (50:50, v/v) prior to HPLC analysis.

Total extractable radioactivity in 50 μL was resolved into parent [^14^C] mesotrione and its polar metabolites by reverse-phase HPLC (Beckman Coulter, System Gold) following the protocol optimized previously in our laboratory (Godar et al., 2015). Reverse-phase HPLC was performed with a Zorbax SB-C18 column (4.6 mm × 250 mm, 5-μm particle size; Agilent Technologies) at a flow rate of 1 mL min^-1^. The radioactivity in the sample was detected using radio flow detector LB 5009 (Berthold Technologies). The whole plant metabolism experiment had three replicates for each treatment and the experiment was repeated. Similarly, the experiment where metabolism of mesotrione in only TL was performed also included three replicates and was repeated.

### RNA Extraction, cDNA Synthesis, and *HPPD* Gene Expression

In this study, the KSR, NER and MSS, KSS, KSS II, KSS III, NES Palmer amaranth plants were not treated with mesotrione, however, adjuvants COC (1% v/v) and AMS (0.85% w/v) were applied to 10–12 cm tall plants. Above ground plant tissue was harvested 24 h after treatment and frozen in liquid nitrogen and stored at -80°C for RNA isolation. The frozen tissue was homogenized in liquid nitrogen using a pre-chilled mortar and pestle to prevent thawing, and transferred 100 mg tissue into a 1.5 mL microcentrifuge tube. Total RNA was isolated using RNeasy Plant Mini Kit (Qiagen Inc., Valencia, CA, USA). The quality and quantity of total RNA was determined using agarose gel (1%) electrophoresis and spectrophotometer (NanoDrop 1000, Thermo Scientific), respectively, and RNA was stored at -80°C.

For cDNA synthesis, 1 μg of total RNA was treated with DNase 1 enzyme (Thermo Scientific, Waltham, MA, USA) to remove any genomic DNA (gDNA). cDNA was synthesized from 1 μg of total RNA using RevertAid First Strand cDNA Synthesis Kit (Thermo Scientific) and was diluted in 1:5 ratio for gene expression study. Quantitative PCR/real-time PCR (qPCR/rtPCR) was used to determine *HPPD* gene expression in all samples. The qPCR reaction mix consisted of 8 μL of SYBR Green mastermix (Bio-Rad Inc., Hercules, CA, USA), 2 μL each of forward and reverse primers (5 μM), and 20 ng cDNA to make the total reaction volume of 14 μL. *HPPD* gene expression was normalized using either *β-tubulin* or carbamoyl phosphate synthetase (*CPS*) as a reference gene. qPCR (CFX96 Touch^TM^ Real-Time PCR Detection System, Bio-Rad Inc.) was performed at 50°C for 2 min, 95°C for 10 min, and 40 cycles of 95°C for 30 s and 60°C for 1 min (Ma et al., 2013). A meltcurve profile was included following the thermal cycling protocol to determine the specificity (no primer dimers, no gDNA contamination, and no non-specific product) of the qPCR reaction. Primer sequences used were: HPPD forward and reverse (F 5′-CTGTCGAAGTAGAAGACGCAG-3′ and R 5′-TACATACCGAAGCACAACATCC-3′); *β-tubulin* forward and reverse (F 5′-ATGTGGGATGCCAAGAACATGATGTG-3′ and R 5′-TCCACTCCACAAAGTAGGAAGAGTTCT-3′); and CPS forward and reverse (F 5′-ATTGATGCTGCCGAGGATAG-3′ and R 5′-GATGCCTCCCTTAGGTTGTTC-3′). The *HPPD*: β-*tubulin* and *HPPD:CPS* expression was determined using the 2ΔC_T_ method, where *C*_T_ is threshold cycle and Δ*C*_T_ is C_T_Reference gene (β-tubulin, or CPS)__- C_T_Target gene (HPPD)__. HPPD gene expression was studied using three biological replicates and three technical replicates for each biological replicate. The experiment was repeated three times and the average value ± standard error of total biological replicates was used to show the expression fold.

### Protein Extraction, SDS–pAGE, and Western Blotting

Above ground plant tissue (0.5 g) from 10 to 12 cm tall Palmer amaranth from KSR, NER and MSS, KSS, KSS II, KSS III and NES was homogenized in liquid nitrogen and added to 20 mL extraction buffer [50 mM Tris-HCl, pH 8, 50 mM NaCl, 1 mM EDTA, 1 mM MgCl_2_, and 0.038 g PMSF, one tablet of Pierce Protease Inhibitor (Thermoscientific), 1 g insoluble PVPP]. The extraction and purification procedure was developed by modifying the methods of Wang et al. (2006) and Wu et al. (2014). In short, homogenates were centrifuged at 4°C, 10 min, 12000 × *g* (Beckman J2-HC centrifuge, USA) and supernatant was collected. One milliliter of TCA (100%) was added to 10 ml of supernatant and incubated for 1 h at 4°C. Samples were centrifuged as before, and the supernatant was discarded. Two milliliter of methanol (100%) was added to the pellet, tubes were vortexed vigorously for 60 s and centrifuged (4°C, 10 min, 12000 × *g*). Supernatant was discarded and acetone (2 ml; 80%) was added to the pellet, vortexed and then centrifuged (4°C, 10 min, 12000 × *g*). Pellet was air dried to remove the remaining acetone and 2 ml phenol (equilibrated with Tris-HCL; pH 8.0, Sigma) was added, vortexed at high speed for 30–60 s and centrifuged (4°C, 10 min, 12000 × *g*) and the supernatant was collected. Proteins were precipitated by adding 2 mL ammonium acetate (0.1 M in methanol) to the supernatant and incubated overnight at -20°C. Next, the sample was centrifuged (4°C, 10 min, 12000 × *g*) and the supernatant was discarded. Pellet was washed with methanol (100%) followed by acetone (80%) and finally air dried. Dried samples were resuspended in 200 μL SDS-Sample buffer and the protein concentration in the extract was determined using the RED 660^TM^ Protein Assay (G-Biosciences).

To resolve proteins in the samples by SDS gel electrophoresis, samples were incubated at 95°C for 5 min. Next, 50 μg of total protein was resolved by electrophoresis on 11% polyacrylamide gel (90 min at 120 V) and transferred to polyvinylidene difluoride (PVDF) membrane (Millipore) at 150 V for 1 h or 30 V overnight. The PVDF membrane was blocked with 5% non-fat dry milk at room temperature for 30 min and then washed three times in TBST. The membranes were incubated with a rabbit polyclonal HPD antibody (Novus biologicals; dilution 1:500) in TBST at 4°C overnight. The membrane was washed three times with TBST and incubated in with donkey anti-rabbit HRP conjugated polyclonal antibody (Jackson Immuno Research Laboratories Inc; dilution 1:50,000) at room temperature for 1 h. After three more washes, membranes were exposed to an HRP substrate solution (Luminata^TM^, Millipore) and image detection was carried out using a G-BOX (Syngene).

### DNA Extraction and *HPPD* Gene Amplification

DNA extraction for *HPPD* gene amplification was performed on the same plant samples used for RNA extraction, cDNA, and *HPPD* gene expression. gDNA was extracted from the frozen leaf tissue (100 mg) using DNeasy Plant Mini Kit (Qiagen) following the manufacturer’s instructions. The quality and quantity of gDNA was determined using agarose gel (0.8%) electrophoresis and spectrophotometer (NanoDrop 1000, Thermo Scientific) and DNA was stored at -20 or -80°C. The following forward and reverse primers (F 5′-CTGTCGAAGTAGAAGACGCAG-3′ and R 5′-TACATACCGAAGCACAACATCC-3′) were used to amplify the *HPPD* gene from Palmer amaranth populations.

### Statistical Analysis

All the experiments were conducted in a completely randomized design, and the data from all experiments were combined for each study before performing statistical analysis as there was no interaction between the experiments and treatments.

Dose-response data (expressed as percentage of the untreated control) were analyzed using ‘drc’ package in R 3.1.2 (Ritz et al., 2015). The three-parameter log-logistic model as shown below was used to show the relationship between herbicide rate and biomass, Y = d/[1+exp{b[log(x) - log(GR_50_)]}] where Y is the response (dry biomass or plant health) expressed as percentage of the untreated control, d is asymptotic value of Y at upper limit, b is the slope of the curve around GR_50_ (the herbicide rate giving response halfway between d and the lower asymptotic limit which was set to 0), and x is the herbicide rate. Resistance index (R/S) was calculated as GR_50_ ratio between the MSS or KSS and the KSR populations.

Absorption and translocation data, expressed as percentage of applied and absorbed, respectively, metabolism data, and qPCR (*HPPD* gene expression) data were analyzed using one-way ANOVA in R 3.1.2 and the means were compared using Tukey’s HSD test. The time course of mesotrione metabolism by MSS and KSR Palmer amaranth populations was fitted with a three-parameter Weibull regression.

## Results

### Mesotrione Dose Response Assay to Determine the Level of Resistance

**FIGURE 1:**
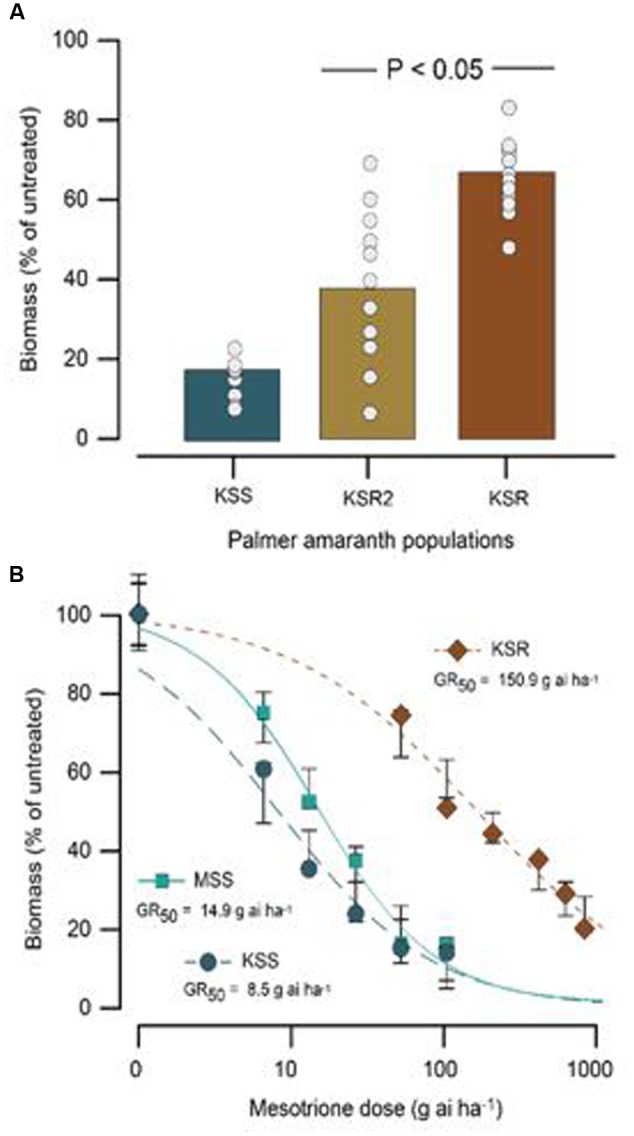
**Response of susceptible (MSS and KSS) and resistant (KSR) Palmer amaranth populations 3 weeks after treatment (WAT) with the herbicide mesotrione. (A)** Individual plant aboveground dry biomass variability in response of KSR compared to the original KSR2 population to 105 g ai ha^-1^ rate of mesotrione, bar indicates the average from 12 individual samples (circles). **(B)** Non-linear regression analysis of aboveground dry biomass of MSS, KSS, and KSR populations at different doses of mesotrione. Symbols are averages of 12 replicates fitted with a three-parameter log-logistic model; model parameters are shown in **Table 1**.

The HPPD-inhibitor-resistant and -susceptible Palmer amaranth populations were derived from different locations. To determine their level of resistance to mesotrione, we conducted dose response assays with these populations. We found a variation in the level of resistance to mesotrione at individual plant level in all populations, especially the KSR2 (**Figure 1A**). This variation is a reflective of genetic variability within and among the populations because the experiments were conducted under controlled environmental conditions (growth chambers) eliminating changes in environmental conditions. Since KSR2 showed extreme variation at 105 g ai ha^-1^ mesotrione, the population was not used further in the dose response analysis. The amount of mesotrione required to reduce plant growth to 50% (GR_50_) 3 WAT was ∼151 g ai ha^-1^ for KSR compared to 15 and 8 g ai ha^-1^ for MSS and KSS, respectively (**Figure 1B**). However, all the surviving resistant individuals showed injury (bleached) symptoms on shoot meristem at all doses of mesotrione and 3 WAT the injured plants did not recover to phenotype of untreated plants, even at low doses of 52.5 g ai ha^-1^ mesotrione. The KSR was 10 and 18 times more resistant compared to MSS and KSS, respectively (**Figure 1B** and **Table 1**). In a different study, the NER Palmer amaranth showed 4- to 14-fold resistance relative to NES in response to mesotrione, tembotrione, and topramezone applications (Sandell et al., 2012).

**Table 1:** Summary parameters describing the response of MSS and KSS (susceptible) and KSR (resistant) Palmer amaranth aboveground dry biomass to rates of mesotrione 3 weeks after treatment (WAT).

Population	Regression parameters^b^		GR_50_^b^	R/S^c^	R/S^d^
	*b*	*d*	g ai ha^-1^		
MSS	1.13	100.8 (4.0)	14.9 (1.7)	1	1.76^∗^
KSS	0.95	100.6 (4.8)	8.5 (1.4)	0.6^∗^	1
KSR	0.69	100.9 (4.1)	150.9 (25.9)	10.1^∗∗^	17.8^∗∗^

*^a^Abbreviations: WAT, weak after treatment; *b*, relative slope around GR_50_; *d*, upper limit of the response; GR_50_, mesotrione rate causing 50% reduction in aboveground dry biomass; R/S, resistance index [ratio of GR_50_ of MSS or KSS (susceptible) and KSR (resistant) populations].*

*^b^Values in parenthesis are ±1 standard error.*

*^c^RS values based on MSS population.*

*^d^RS values based on KSS population.*

*^∗,∗∗^R/S is significantly greater than 1 at *P* < 0.001, *P* = 0, repectively.*

*The response was fitted with a three-parameter log-logistic model; fitted curves are shown in **Figure 1B**.*

### Absorption of [^14^C] Mesotrione and Translocation of [^14^C] Compounds

**FIGURE 2:**
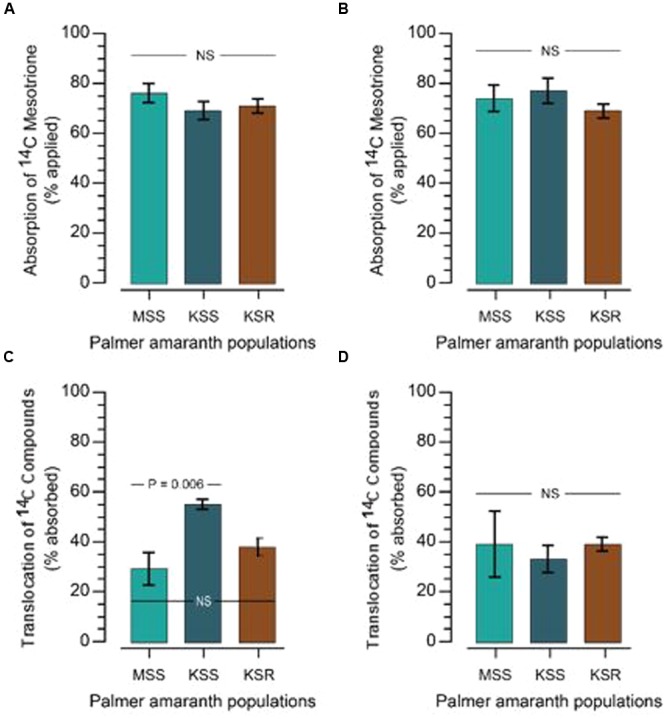
**[^14^C] mesotrione absorption and translocation in resistant and susceptible Palmer amaranth populations. (A,B)** Absorption of [^14^C] mesotrione; **(C,D)** translocation of [^14^C] compounds in resistant (KSR) and susceptible (MSS and KSS) Palmer amaranth populations. Absorption and translocation in the plant was measured at 48 **(A,C)** and 72 **(B,D)** hours after treatment (HAT) with liquid scintillation spectrometry (LSS). Data were analyzed using one-way ANOVA and the means were compared using Tukey’s HSD test. Error bars represent standard error of means (*n* = 6) at each time point. NS, non-significant at α = 0.05.

The resistance/higher tolerance to mesotrione and other HPPD-inhibiting herbicides can arise through a variety of mechanisms. First, we tested if there is a difference in absorption by measuring how much [^14^C] mesotrione was absorbed by the resistant and susceptible plants. Absorption of [^14^C] mesotrione in KSR at 48 and 72 HAT was 71 and 69% (as % of total applied), which was not significantly different from the susceptible populations (76 and 74% in MSS and 69 and 77% in KSS, at 48 and 72 HAT, respectively; **Figures 2A,B**, *P* > 0.05).

**FIGURE 3:**
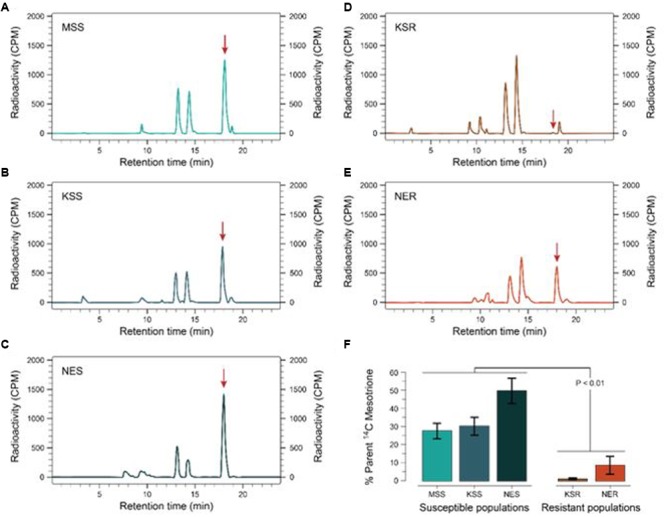
**Metabolism of [^14^C] mesotrione in resistant and susceptible Palmer amaranth populations harvested at 24 HAT.** Reverse-phase HPLC chromatograms of plants treated with [^14^C] mesotrione and harvested **(A)** MSS **(B)** KSS **(C)** NES **(D)** KSR, and **(E)** NER. Peak retention time around 18.1 min is the mesotrione (input) and other peaks 13.1 and 14.3 min are the major metabolites of [^14^C] mesotrione. **(F)** Represents the amount of [^14^C] mesotrione input remaining as percentage of total in the resistant (KSR and NER) and susceptible populations (MSS, KSS and NES) 24 HAT. Data were analyzed using one-way ANOVA and the means were compared using Tukey’s HSD test. Error bars represent the standard error of means of 6–9 biological replicates.

Resistance can be derived if the plants have reduced translocation of the herbicide. Since, [^14^C] mesotrione after application can be translocated to ATL, BTL or roots or stay in TL as [^14^C] mesotrione or its metabolites and it is difficult to separate specific [^14^C]’s, it is more appropriate to say translocation of [^14^C] compounds. Data analysis showed no significant differences in the translocation of [^14^C] compounds to ATL or BTL from TL at 48 HAT between resistant or susceptible populations. KSR (37% expressed as % of total [^14^C] mesotrione absorbed) showed translocation that was in between both susceptible populations MSS (29%) and KSS (55%) populations (**Figure 2C**, *P* > 0.05). This suggests that there is an underlying genetic variation in the ability of Palmer amaranth to translocate mesotrione that does not correlate with resistance. This variation is likely responsible for the significant difference we observed in the translocation of [^14^C] mesotrione between the MSS and KSS. Furthermore, the significant difference disappeared at 72 HAT where the KSR, MSS and KSS had 39, 33, and 39%, respectively, of [^14^C] mesotrione translocated from the TL, to the above and below treated plant parts (**Figure 2D**, *P* > 0.05). In addition, because of rapid metabolism of mesotrione in resistant plants (**Figure 3**) it was not possible to say whether there were any differences in the translocation of mesotrione between resistant and susceptible Palmer amaranth. However, assuming that the major metabolites of mesotrione move in a similar way as the parent molecule, translocation appears to be similar. Thus, neither difference in mesotrione absorption nor translocation contributed substantially to mesotrione resistance in KSR Palmer amaranth.

### Metabolism of [^14^C] Mesotrione

**FIGURE 4:**
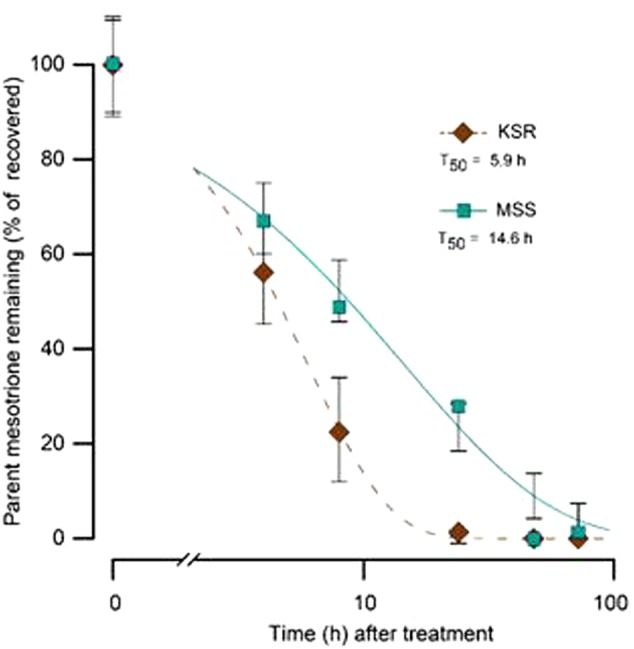
**The time course of [^14^C] mesotrione metabolism (T_50_) in the treated leaves MSS (susceptible) and KSR (resistant) Palmer amaranth populations across 4, 8, 16, 24, 48, and 72 HAT.** Error bars represent the standard error of means of 6–9 biological replicates.

Some weeds also have been shown to acquire resistance by increasing their ability to metabolize specific herbicides. To test for a role of metabolism based resistance in the KSR population, we measured how much [^14^C] mesotrione was metabolized into other polar compounds over time. The input [^14^C] mesotrione resolved at peak retention time of about 18.1 by reversed-phase HPLC with no other peaks observed (data not shown). This indicates that peaks at 13.1 and 14.3 retention times observed in plant lysates are products derived from mesotrione metabolism (**Figure 3**). These peaks gradually increased with decrease in input [^14^C] mesotrione in all the populations indicating that the metabolites might be hydroxylated products of mesotrione (Ma et al., 2013). To determine the % of mesotrione remaining, we quantified the amount of radioactivity of the 18.1 peak as fraction of total radioactivity. As early as 4 HAT we observed significant differences with more than 70% of input parent [^14^C] mesotrione still being detected in susceptible samples, while in KSR plants ∼50% of parent [^14^C] mesotrione was metabolized (data not shown). At 24 HAT, KSR, and NER metabolized much more parent compound (>90%) compared to MSS, KSS and NES (**Figures 3A–E**) (*P* < 0.01), which still showed about 28, 30, and 50%, respectively, of parent [^14^C] mesotrione. This amount of mesotrione was sufficient to injure the plant and subsequently kill the susceptible plants 3 WAT. The half-life T_50_ is the amount of time taken for 50% of the parent input [^14^C] mesotrione to degrade or metabolize inside the plant through enzymatic transformation. It was found that T_50_ for MSS and KSR was 14.6 and 5.9 h, respectively, indicating that KSR metabolizes the mesotrione 2.5 times faster compared to the MSS (**Figure 4** and **Table 2**). These metabolism data indicate that mesotrione metabolism is contributing significantly to the resistance in Palmer amaranth. However, interestingly, both resistant and susceptible Palmer amaranth populations were able to completely metabolize parent [^14^C] mesotrione by 48–72 HAT (data not shown) further suggesting that rapid metabolism alone may not solely conferring resistance to mesotrione in KSR or NER.

**Table 2:** Summary parameters describing the time course of mesotrione metabolism by MSS (susceptible) and KSR (resistant) Palmer amaranth populations.

Population	Regression parameters^a^		T_50_^a^	R/S
	*b*	*d*	*h*	
MSS	0.74	99 (5.0)	14.6 (1.9)	1
KSR	1.33	100 (5.0)	5.9 (0.5)	2.45^∗^

*b, relative slope around T_50_; *d*, upper limit of the response; T_50_, time taken to metabolize 50% of recovered mesotrione; R/S, resistance index [ratio of T_50_ of MSS (susceptible) and KSR (resistant) populations].*

*^a^Values in parenthesis are ±1 standard error.*

*^∗^R/S is significantly greater than 1 at *P* < 0.01.*

*The response was fitted with a three-parameter Weibull regression; fitted curves are shown in **Figure 4**.*

### Analysis of *HPPD* Gene Expression

**FIGURE 5:**
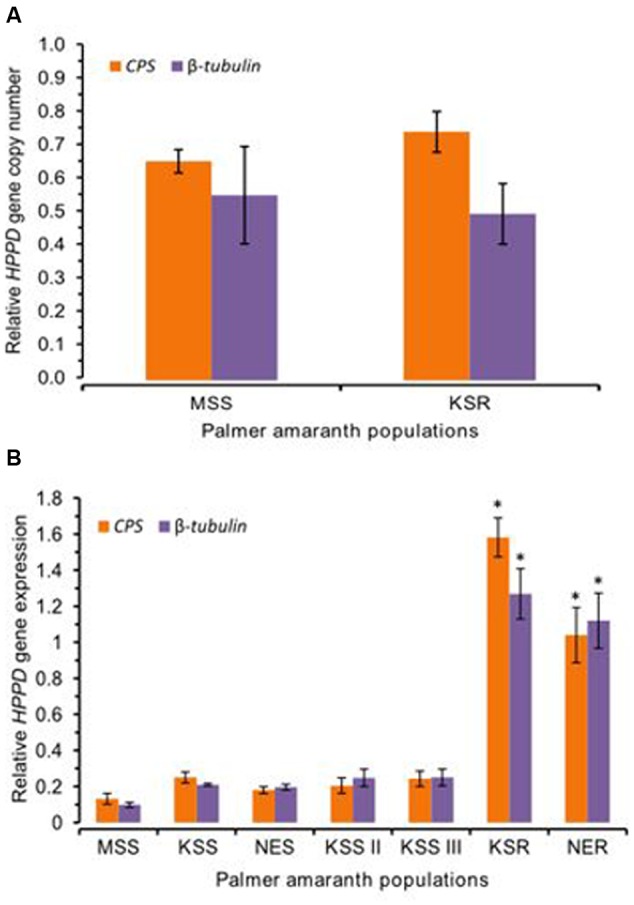
**4-hydroxyphenylpyruvate dioxygenase (*HPPD*) gene amplification and expression. (A)** *HPPD* genomic copy number in MSS (susceptible) and KSR (resistant) Palmer amaranth relative to two reference genes *CPS* and β-*tubulin*. Error bars represent the standard error of means of 12 biological replicates. **(B)** The amount of *HPPD* gene expression in the susceptible Palmer amaranth populations (MSS, KSS, KSS II, KSS III, and NES) and resistant Palmer amaranth populations (KSR and NER). The amount of *HPPD* gene expression was normalized to the corresponding level of two reference genes, β-*tubulin* and *CPS*. Data were analyzed using one-way ANOVA and the means were compared using Tukey’s HSD test. Bars represent the means ± SE of 6–9 biological replicates. Asterisks above error bars represent significant difference in *HPPD* gene expression compared to corresponding to each susceptible population MSS, KSS, KSS II, KSS III, or NES at α = 0.05.

We tested for possible mutation or amplification of the *HPPD* gene conferring resistance to mesotrione in Palmer amaranth. However, our data did not show any mutations or amplification of the *HPPD* gene in this population (**Figure 5A**). Therefore, we hypothesized that, in addition to rapid metabolism, increased expression of the *HPPD* gene may possibly contribute to mesotrione resistance in KSR or NER. To test this idea, mRNA levels of the *HPPD* gene in all mesotrione-resistant and -susceptible Palmer amaranth individuals were determined. Since genetic variation as well as variability in the degree of sensitivity to mesotrione exists, there was 1- to 2.5-fold variation in *HPPD* gene expression among the five susceptible populations (MSS, KSS, KSS II, KSS III, and NES). *HPPD* mRNA levels in KSR and NER (normalized against β*-tubulin* and *CPS)* was at least 12-fold and 8- to 12-fold higher, respectively, compared to MSS (**Figure 5B**, *P* < 0.001). When compared to the other four susceptible populations, KSS, KSS II, KSS III, and NES, *HPPD* gene expression relative to β*-tubulin* or *CPS* was least 4- to 9-fold more in KSR and NER (**Figure 5B**, *P* = 0.001). These data indicate that the basal mRNA levels for HPPD are strongly upregulated in resistant populations. This increase in *HPPD* gene expression is likely to an important role in the initial response of resistant Palmer amaranth when mesotrione is applied.

### HPPD Protein Expression in Mesotrione-Resistant Palmer Amaranth

**FIGURE 6:**
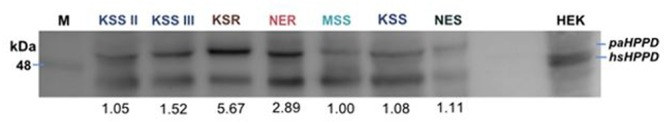
**Protein lysates of indicated populations were resolved by 11% SDS–PAGE and immunoblotted for the presence of HPPD in mesotrione-susceptible (MSS, KSS, NES, KSS II, and KSS III) and -resistant (KSR and NER) Palmer amaranth populations using a rabbit polyclonal antibody against human HPD antibody at 50 μg protein concentration.** Last lane contains lysate derived from HEK 293 cells (human embryonic kidney cells) and was used as a positive control. Blot shows one individual plant from each population at 50 μg. M represents the marker. The blots were also quantified and MSS is normalized to 1 and other populations were calculated relative to MSS (numbers shown below the blot for each lane).

To investigate whether the *HPPD* mRNA transcript abundance correlates with increased HPPD protein levels, we next conducted immunoblot analysis. No antibody is available against Palmer amaranth HPPD; however, *Amaranthus* HPPD is 35% identical with human HPPD. Therefore, we used a human HPPD antibody to test if there is cross-reactivity with the Palmer amaranth *HPPD* protein. As shown in **Figure 5**, the antibody recognized HPPD in human cell lysates (HEK lysate). In the Palmer amaranth lysate, a protein with molecular weight of about 48 kDa was detected, which is consistent with the anticipated size of *Amaranthus* HPPD. The protein could be detected in both susceptible and resistant Palmer amaranth populations, however, KSR or NER lysates showed more HPPD protein as compared to MSS, KSS, KSS II, KSS III, or NES lysates at 50 μg protein concentrations (**Figure 6**). The differences in the HPPD protein between the KSR and NER can be explained because plants in the KSR population are more uniform with their response to mesotrione, while NER is a field collected population segregating and exhibiting variation in plant to plant response to mesotrione application. Since a polyclonal HPPD antibody was used, non-specific and cross hybridization occurred due to the cross-reactivity of the antibody with other proteins in the sample. In all, our data indicate that the increased mRNA levels observed in the resistant populations are translated into increased protein levels.

## Discussion

4-hydroxyphenylpyruvate dioxygenase-inhibiting herbicides are relatively new group of herbicides which effectively control a broad spectrum of broadleaf and some grass weeds. Mesotrione is a triketone developed for pre- and post-emergence control of many broadleaf weeds along with some grass weeds in corn. To date, only two weeds species, belonging to the same botanical family, Amaranthaceae, have evolved resistance to HPPD-inhibitors, namely waterhemp and Palmer amaranth (Hausman et al., 2011; Thompson et al., 2012; Heap, 2017). Plant species can evolve resistance to herbicides essentially via two main mechanisms, (a) non-target-site based involving decreased absorption, reduced translocation and/or enhanced metabolism of herbicides and (b) target-site based as a result of mutations in the target gene or increased levels of the target protein, enabled through gene amplification or transcriptional upregulation. Absorption and translocation of mesotrione was similar for mesotrione-resistant and -susceptible Palmer amaranth populations in this research (**Figure 2**) and, thus, did not appear to contribute to resistance. However, greater sensitivity observed in KSS (GR_50_ 8 g ha^-1^) in the dose response assay compared to MSS (GR_50_ 15 g ha^-1^) might have resulted from increased translocation of mesotrione (**Figure 2C**). The absorption of [^14^C] mesotrione in Palmer amaranth is consistent and corresponds to the mean absorption of radio labeled mesotrione across different time points as reported in waterhemp population from IL (Ma et al., 2013). Once absorbed, these herbicides generally translocate via both xylem and phloem (Mitchell et al., 2001; Beaudegnies et al., 2009) to other parts of the plant. However, the translocation of [^14^C] mesotrione data showed no significant differences contributing to mesotrione resistance.

Plants can detoxify both exogenous and endogenous compounds through a large family of enzymes known as cytochrome P450 monooxygenases. However, the degree to which each plant can metabolize and degrade xenobiotic chemicals is a major contributor to their survival and in the evolution of resistance. For example, crops like corn, wheat, rice, and sugarcane have a natural tolerance to several groups of herbicides (e.g., HPPD-, ALS-inhibitors) conferred by cytochrome P450 detoxification mechanism (Kreuz et al., 1996; Mitchell et al., 2001). Enhanced detoxification, likely by cytochrome P450 monooxygenases as the mechanism of mesotrione resistance, has been reported in waterhemp population from IL (Ma et al., 2013). The data presented here suggest that Palmer amaranth resistance to mesotrione results, primarily, from the ability to rapidly metabolize this herbicide (**Figure 3**). Our data shows a strong correlation between the rate of mesotrione degradation and the degree of susceptibility or resistance. Resistant Palmer amaranth (KSR) was able to detoxify 50% of mesotrione (T_50_ 5.9 h; **Figure 4**) in a short time compared to corn (T_50_ 11.9 h) and waterhemp (T_50_ 12 h) (Ma et al., 2013). Similarly, waterhemp susceptible to mesotrione required about 30 h (T_50_) which is about two times slower than susceptible Palmer amaranth. However, our data also suggest that the susceptible individuals also completely metabolize mesotrione by 48–72 HAT indicating that detoxification of mesotrione alone may not be the only mechanism of resistance in Palmer amaranth. In weeds, oxidation, hydroxylation, or dealkylation of different herbicides, by cytochrome P450s has been reported to be one of the major non-target-site mechanisms confirming resistance to herbicides in both broadleaf and grass weed species (Powles and Yu, 2010).

Recently a rice cytochrome P450 gene, CYP72A31 has been identified to confer resistance to ALS-inhibiting herbicides in both rice and *Arabidopsis* (Saika et al., 2014). Previously Pan et al. (2006) reported involvement of rice CYP81A6 in imparting resistance to PS II- and ALS-inhibiting herbicides. Furthermore, when wheat CYP71C6v1 cDNA was cloned and expressed in yeast, ALS inhibiting herbicides were metabolized via phenyl ring hydroxylase (Xiang et al., 2006). Transcriptomic analysis of diclofop-resistant rigid ryegrass (*Lolium rigidum*) revealed involvement of three Cytochrome P450 genes, a nitronate monooxygenase (NMO), three GST, and a glucosyl transferase (GT) in detoxification of diclofop (Gaines et al., 2014). However, the specific role of cytochrome P450s in detoxification of mesotrione is unknown and might not suffice to induce agriculturally significant resistance. Especially, since it seems to only be temporal difference, as all populations are able to fully metabolize mesotrione in 48 h. Though primary, faster degradation of mesotrione alone may not be significant for resistance of Palmer amaranth at recommended field rates or higher.

In addition to the non-target mechanism of rapid detoxification of mesotrione, the target-site based resistance mechanism(s) such as mutation or amplification of HPPD were also tested in our KSR populations. Sequencing of the *HPPD* gene did not show any mutations (unpublished) or amplification in this population. On the other hand, we found a significant increase in *HPPD* gene and protein expression (**Figures 5B, 6**) in mesotrione-resistant populations, suggesting that the resistant plants have a sufficiently high amount of HPPD enzyme available for maintaining the function of carotenoid biosynthetic pathway even when exposed to field rate of mesotrione. Biochemically, mesotrione and other HPPD-inhibiting herbicides act as competitive inhibitors of the HPPD enzyme involved in the conversion of 4-hydroxyphenylpyruvate (HPP) to 2,5-dihydroxyphenylacetate (homogentisate) (Beaudegnies et al., 2009). In the model plant, *Arabidopsis thaliana*, constitutive over expression of *HPPD* that was 10-fold higher than the wild type plants showed increased tolerance to sulcotrione, a triketone herbicide (Tsegaye et al., 2002). Similarly, heterologous expression of barley *HPPD* in tobacco also resulted in 10-fold higher resistance to sulcotrione (Falk et al., 2003).

Interestingly, a combined resistance through detoxification and target site upregulation has been observed to insecticides in mosquitoes. Here, it has been reported that the insects upregulate metabolic enzymes, esterases, GSTs, or cytochrome P450 monooxygenases through changes/mutations in the *cis*/*trans*-acting elements, gene regulation or via amplification of the genes encoding these enzymes (Xianchun et al., 2007). For example, in southern house mosquito (*Culex quinquefasciatus*), *CYP9M10* is overexpressed to 260-fold higher in a pyrethroid-resistant compared to a susceptible strain via two mechanisms. Two copies of a large fragment of ∼100 kb containing the *CYP9M10*, flanked by MITE (a transposable element) of about 0.2 kb upstream of duplicated copies were found. Since only two copies of this cytochrome cannot explain the 260-fold upregulation, the *cis*-acting and promoter regions were sequenced and it was discovered that there was a *cis*-acting mutation which mediated increased expression (Itokawa et al., 2010). To our knowledge, this is the first case of Palmer amaranth that naturally evolved mesotrione resistance because of increased target-site gene expression without gene amplification. Increased gene expression can occur without increase in gene copies via changes in the *cis* or *trans*-acting elements, alterations in the promoter region of the gene or post-transcriptional mechanisms that regulate gene expression (Gallie, 1993; Carino et al., 1994; Chung et al., 2007). Glyphosate-resistant junglerice (*Echinochloa colona*) showed enhanced basal *EPSPS* activity of 1.4-fold compared to the susceptible plants, possibly through such changes (Alarcón-Reverte et al., 2015). Similar molecular process could be involved in *A. palmeri* that confer resistance to mesotrione. Experiments are in progress in our laboratory to investigate the genetics of non-target-site based (metabolism) and target-site based (increased *HPPD* gene expression) resistance to mesotrione using forward genetics approach in our Palmer amaranth population.

In addition to herbicide selection pressure, availability of extensive genetic variability, high growth rate and fecundity, adaptation to wide ecological conditions in Palmer amaranth (Knezevic et al., 1997), metabolic resistance and increased *HPPD* gene expression provides an adaptive advantage to survive and spread under diverse environmental stresses. However, the fitness of such herbicide-resistant Palmer amaranth is not known and investigation of fitness costs associated with the resistance trait can help predict the dynamics of evolution and spread of mesotrione resistance in other populations. Furthermore, transcriptome analysis of mesotrione-resistant Palmer amaranth with multiple mechanisms will be a valuable genetic resource: (a) to identify and characterize the precise role of specific cytochrome P450s and other target and non-target genes in mesotrione resistance and (b) in the research and development of novel herbicides and herbicide tolerant crops.

The mesotrione-resistant Palmer amaranth populations used in this study are also resistant to atrazine and chlorsulfuron (ALS-inhibitor), two widely used herbicides in corn production. In general, HPPD-inhibitors are a viable option to manage weeds that are resistant to PS-II and ALS-inhibitors in corn. As Palmer amaranth is a troublesome weed in corn, evolution of resistance to HPPD-inhibitors in this weed will leave fewer herbicide options for management. As no new herbicide modes of action have been discovered in more than two decades, it is increasingly important to effectively and efficiently use currently available herbicides for sustainable agricultural production. More importantly, the non-target-site based mesotrione resistance in Palmer amaranth may exhibit cross resistance to other known and unknown herbicides that are yet to be discovered. Hence, the weed management strategies in regions with Palmer amaranth and other weeds should include diversified tactics to effectively prevent evolution and spread of multiple herbicide resistance.

## Author Contributions

MJ conceived and supervised the work. SN designed, planned and performed the experiments and analyzed the data. AG performed the statistical analysis and interpretation of the results. PW and JR contributed in western blotting experiment and protein expression analysis. CT provided the seed, and DP and CT revised the manuscript critically.

## Conflict of Interest Statement

The authors declare that the research was conducted in the absence of any commercial or financial relationships that could be construed as a potential conflict of interest. The reviewer PTFM and handling Editor declared their shared affiliation, and the handling Editor states that the process nevertheless met the standards of a fair and objective review.
